# Kraft Lignin-Based Polyurethane with GVL: A Sustainable Coating Alternative for Recycled Linerboard

**DOI:** 10.3390/polym18010118

**Published:** 2025-12-31

**Authors:** Julia C. Figueiredo, Roberto C. C. Lelis, Rosane N. Castro, Fernando J. B. Gomes, Ericka F. A. Redmond, Biljana M. Bujanovic

**Affiliations:** 1 Forest Products Department, Forestry Institute, Universidade Federal Rural do Rio de Janeiro, BR-465 km 7, Seropédica 23897-000, RJ, Brazil; lelis@ufrrj.br (R.C.C.L.); nora@ufrrj.br (R.N.C.); fernandogomes@ufrrj.br (F.J.B.G.); 2 Department of Chemical Engineering, College of Environmental Science and Forestry, State University of New York, 1 Forestry Drive, Syracuse, NY 13210, USA; eredmond@esf.edu; 3 Forest Products Laboratory, U.S. Department of Agriculture-Forest Service, 1 Gifford Pinchot Drive, Madison, WI 53726, USA

**Keywords:** paper packaging, linerboard, coating, lignin-based polyurethane, green solvents, barrier and mechanical properties, repulpability, biodegradation

## Abstract

Food packaging is the largest segment of the global plastics market, yet its low degradability and limited performance in preserving perishable goods highlight the need for more sustainable alternatives. This study investigates the use of industrial softwood kraft lignin, a renewable polyol, and γ-valerolactone (GVL), an excellent green lignin solvent, to synthesize bio-based polyurethane (PU) coatings for recycled linerboard. PU was synthesized with hexamethylene diisocyanate (HDI), GVL, and 1,4-diazabicyclo[2.2.2]octane (DABCO) as a catalyst and applied to recycled linerboard (166.6 g/m^2^) at three coating weights: 13.5, 16.5, and 23.5 g/m^2^. The coating enhanced water resistance, as shown by the reduced water vapor transmission rate (WVTR) and Cobb_1800_ values. Oil resistance was also significantly improved, reaching a Kit rating of 11 at the highest coating weight. Mechanical performance was maintained or enhanced, with increases in ring crush strength (RCT) and tensile index. These findings confirm the effectiveness of lignin-based PU in improving both the barrier and mechanical properties of packaging paper. Additionally, this approach presents an environmentally responsible alternative to petroleum-based coatings, adding value to lignin as a byproduct of the pulp and paper industry and supporting the transition toward more circular and sustainable packaging materials.

## 1. Introduction

Food packaging currently dominates the global plastics market, with widespread use driven by demand in the food sector [1,2,3]. However, the poor degradation of petroleum-based plastics, combined with their limited ability to preserve perishable products, underscores the need for alternative materials. Recent efforts have focused on the development of bio-based plastics that combine natural degradability with high-performance protective properties [3]. These biopolymer-based plastics can serve as functional coatings for paper packaging, improving its barrier properties.

Paper is widely used in packaging because it is biodegradable, flexible, and cost-effective. However, its porous structure and hydrophilic nature result in poor barrier and sealing performance, limiting its overall effectiveness [4,5,6]. To address these limitations, fossil-based polymer coatings are widely employed, but their use diminishes the environmental and sustainable benefits of paper. This has led to a growing interest in bio-based alternatives [4]. In the search for more sustainable alternatives, polymers that can be based on renewable constituents, such as polyurethanes (PUs), have gained attention [7,8,9,10,11]. PUs, formed by the reaction of renewable polyols and isocyanates, offer tunable properties and are used in a wide range of applications, including packaging [12,13,14].

Among renewable polyols, lignin, particularly kraft lignin, stands out due to its aromatic structure and abundance of hydroxyl groups, making it a promising substitute for petrochemical polyols in PU synthesis [15,16,17]. Despite this potential, challenges such as low solubility, structural heterogeneity, and limited accessibility of reactive hydroxyl groups hinder the direct use of lignin without chemical modification [18]. Several studies have reported chemical modification or fractionation of lignin to improve its reactivity in polyurethane synthesis [19,20,21]. Alternative organic solvents have also been explored to facilitate these reactions and improve the utilization of kraft lignin in its original form [10,22,23]. However, the toxicity of many such solvents raises concerns regarding environmental safety and their suitability for sustainable material production.

The search for greener and safer solvents has highlighted γ-valerolactone (GVL) as a promising solvent for biomass processing, capable of effectively dissolving and depolymerizing lignin under mild conditions [24]. This biomass-derived solvent is widely utilized in the production of additives for food and fuel applications [25]. GVL is non-toxic, has low volatility, and is completely miscible with water without forming an azeotrope [26].

From an environmental standpoint, GVL is considered a green solvent, derived from the hydrogenation and cyclization of levulinic acid obtained from lignocellulosic biomass. It exhibits high biodegradability, low toxicity, and excellent thermal stability (boiling point of ~207 °C and melting point of −31 °C), reinforcing its potential as a substitute for fossil-based solvents in industrial processes [27,28]. The effectiveness of GVL in dissolving lignin is attributed to its strong molecular affinity for lignin, as evidenced by a low relative energy difference (RED = 0.83) based on Hansen’s solubility parameter theory, demonstrating greater compatibility than conventional solvents such as ethanol (RED = 0.99) and acetone (RED = 1.21) [29,30]. The superior compatibility between lignin and GVL stems from GVL’s intermediate polarity and high dielectric constant (ε ≈ 36.5), which promote interactions with lignin’s hydroxyl and aromatic groups [27]. Based on these advantageous properties, GVL has recently been investigated for its application in both biorefinery operations and lignin valorization processes.

Wu et al. (2022) [25] studied the production of lignin nanoparticles (LNPs) from wheat straw black liquor using nanoprecipitation with GVL. These nanoparticles, well-dispersed within the polyurethane (PU) matrix, acted both as reinforcing fillers and hydrogen bond donors, significantly improving the mechanical properties of the PU films. The incorporation of LNPs led to an increase in tensile strength and elongation at break compared to neat PU. Additionally, LNPs conferred enhanced hydrophobicity, UV-blocking capacity, and hydrothermal stability [25].

Granatier et al. (2023) [26] proposed an innovative GVL-based biorefinery concept in which birch wood was fractionated using GVL, allowing for efficient recovery of all major biomass components. A key focus was the valorization of GVL-solubilized lignin, structurally analogous to Alcell™ lignin, for the synthesis of bio-based polyhydroxyurethane (GVL-PHU). Acting as a reactive polyol, this lignin was incorporated into polyurethane networks, yielding materials with tunable mechanical performance and thermal degradation temperatures reaching up to 440 °C. FTIR and TGA analyses confirmed the formation of urethane linkages and enhanced thermostability [26].

GVL can be employed in polyurethane synthesis through condensation reactions with various monomers. Adjusting precursor structures enables precise tuning of the material’s thermal and mechanical properties [24]. Given softwood kraft lignin’s potential as a bio-based polyol and GVL’s advantages as a sustainable solvent, this study aimed to synthesize polyurethane (PU) films from lignin solubilized in GVL and evaluate their structural and functional performance as paper coatings. The lignin-based PU was characterized by Fourier Transform Infrared Spectroscopy (FTIR) and Thermogravimetric Analysis (TGA) to investigate its chemical structure and thermal behavior prior to application on paper surfaces. The mechanical performance and barrier properties of the coated paper were then assessed to determine its suitability for sustainable packaging applications.

## 2. Materials and Methods

### 2.1. Material

Recycled linerboard paper (grammage and thickness of 166.6 g/m^2^ and 251 µm, respectively), sourced from a paper mill, was used as the base substrate for coating. Technical softwood kraft lignin was supplied by a pulp mill. Both materials are commercial and were donated to the State University of New York–College of Environmental Science and Forestry (SUNY-ESF). The bio-based polyurethane was synthesized using hexamethylene diisocyanate (HDI), gamma-valerolactone (GVL) and 1,4-diazabicyclo[2.2.2]octane (DABCO), all purchased from Fisher Scientific. The research was conducted at the Department of Chemical Engineering at SUNY-ESF and at the Universidade Federal Rural do Rio de Janeiro (Lignocellulosic Biorefinery Laboratory).

### 2.2. Methods

#### 2.2.1. Lignin Characterization

The molecular weight distribution of lignin was measured using an Agilent Gel Permeation Chromatography (GPC) SECurity 1200 system (Agilent Technologies). Prior to GPC analysis, kraft lignin was acetylated with acetic anhydride/pyridine (1:1) [31], dissolved in tetrahydrofuran (THF), and filtered through 0.45 μm PTFE filters. The insoluble and soluble lignin contents were determined according to NREL/TP-510-42618, and the ash content of kraft lignin was measured following NREL/TP-510-42622.

Phosphorus Nuclear Magnetic Resonance (^31^P NMR) analysis was performed to quantify and identify hydroxyl groups in kraft lignin using a Bruker AVANCE 500 MHz NMRspectrometer (Bruker BioSpin GmbH & Co. KG) equipped with a broadband cryogenically cooled probe. For sample preparation, vacuum-dried lignin (20 mg) was dissolved in 500 μL of an internal standard solution composed of endo-N-hydroxy-norbornene-2,3-dicarboximide (NHND) and chromium acetylacetonate [Cr(acac)^3^] in pyridine/deuterated chloroform (CDCl_3_) (1.6:1, *v*:*v*). After complete dissolution, 100 μL of 2-chloro-4,4,5,5-tetramethyl-1,3,2-dioxaphopholane (TMDP) was added as a phosphitylation reagent. The ^31^P NMR experiment was conducted immediately after sample preparation. Acquisition parameters included a spectral width of 100 ppm, an acquisition time of 0.8 s, a relaxation delay of 10 s, and 64 scans [32].

Fourier Transform Infrared Spectroscopy, PerkinElmer Frontier FT-IR/NIR (PerkinElmer Inc.), was used to record the spectra of lignin. Samples were dried at 40 °C in a vacuum oven before analysis. FTIR spectra were recorded at room temperature using 32 scans in the range of 500–4000 cm^−1^.

#### 2.2.2. Lignin-Based Polyurethane Synthesis

As kraft lignin is solid, it was solubilized to facilitate polyurethane formation. Briefly, 9 g of softwood kraft lignin and 0.03 g of DABCO were dissolved in 16 g of GVL under constant stirring for 15 min. After this period, 6 g of HDI was added, and stirring continued for an additional 15 min. The reaction mixture was then left to stand for approximately 3 h until it reached a suitable viscosity for coating, preventing penetration into the paper substrate. The reaction was conducted in three stages: initially, it proceeded for one hour in a fume hood at approximately 20 °C; subsequently, it was heated in an oven at 105 °C for 10 min; finally, it continued at room temperature (~20 °C) for two hours. A control reaction without kraft lignin was performed under the same conditions, as GVL could react with isocyanate. The synthesized polyurethanes with and without kraft lignin were designated as KL-PU and Control-PU, respectively.

To confirm polyurethane formation, FTIR analysis was performed using 32 scans in the range of 500–4000 cm^−1^ at room temperature. Thermogravimetric analysis (TGA) was carried out using a T550 TGA (Waters TM TA Instruments) to evaluate the thermal stability and mass loss of the materials. PU samples (~8 mg) were heated from room temperature to 600 °C at a rate of 10 °C/min under a nitrogen atmosphere.

#### 2.2.3. Coating Application

Approximately three and a half hours after the initiation of polymerization, the reaction mixture reached a polymerization stage suitable for coating application, allowing for uniform deposition on the paper surface without penetration through the sheet thickness. The coating was applied in two layers using an automatic coater, K Control Coater (RK PrintCoat Instruments Ltd.), with variations in bar thickness and application speed. Only KL-PU was applied to the paper surface, as it formed a continuous and uniform film under the selected application conditions. In contrast, the Control-PU formulation did not exhibit adequate film-forming behavior, either penetrating through the paper structure or undergoing premature curing during application, which prevented uniform surface coverage.

The coating was applied at three different dry coat weights (13.5, 16.5 and 23.5 g/m^2^) on recycled linerboard (166.6 g/m^2^) to investigate the effect of coating quantity on the physical and barrier properties of this porous substrate. These coat weights were selected to represent a practical and industrially relevant range for surface treatments on paper-based packaging materials, balancing coating effectiveness, processability, and cost. Lower coat weights (<13 g/m^2^) may be insufficient to form a continuous and effective barrier on highly porous substrates such as recycled linerboard. Conversely, coat weights above 25 g/m^2^ are less commonly used industrially due to challenges related to longer drying times, increased material consumption, and potential negative impacts on flexibility and adhesion.

#### 2.2.4. Paper Characterization

All coated and uncoated papers were analyzed using several tests, as specified in various TAPPI and ASTM standards shown in Table 1.

#### 2.2.5. Scanning Electron Microscopy (SEM)

Scanning Electron Microscopy (SEM) was performed using a Hitachi TM3000 equipment (Hitachi, Ltd.) at 100×, 300× and 400× magnification, 15,000 Volts and low vacuum. Prior to analysis, the paper samples were acclimatized in a room with controlled temperature and humidity (23 ± 1 °C and 50 ± 2% RH).

#### 2.2.6. Biodegradability

The ASTM D5338-15 standard [43] was adapted for the biodegradability test. Organic compost was supplied and fully characterized by COMLURB (Municipal Urban Cleaning Company, Rio de Janeiro, Brazil). Paper samples, with and without coating, were cut into 2 × 2 cm, and each specimen was weighed after acclimatization (23 ± 1 °C and 50 ± 2% RH). To prepare the system, 130 g of organic compost with 50% moisture and pH 7.4 was weighed. Three specimens were added to each system and stored in a temperature-controlled incubator (58 ± 2 °C) for six weeks. Each week, the paper samples were cleaned and re-acclimatized (23 °C and 50% RH) for 72 h before weighing. The percentage mass loss of each sample was then calculated gravimetrically. SEM analyses of the papers were conducted before and after biodegradation.

For the biodegradability assessment, only the KL-PU (23.5) was selected as a representative case. This sample was chosen based on two main criteria: (i) it represents a conservative “worst-case scenario” from an environmental perspective, as it contains the highest amount of polyurethane, and (ii) it exhibited the most significant improvements in barrier performance, including water vapor and oil resistance. Therefore, evaluating biodegradability under this condition ensures that the environmental feasibility of the lignin-based PU coating is assessed at both its highest functional performance and maximum polymer loading. Coatings with lower coating weights are expected to show comparable or improved biodegradation behavior.

#### 2.2.7. Repulpability

The repulpability test was performed according to ASTM D5999-96 [44] and APCO’s Standard Pulpability Methodology [45]. Coated and uncoated samples were cut into 2.5 × 2.5 cm pieces and hydrated overnight (approximately 12 h). The disintegration process was conducted in a TAPPI/British disintegrator under controlled conditions: temperature of 45 °C, pH adjusted to ~7.5, consistency of 1.5 wt%, and a total of 40,000 revolutions. After disintegration, the pulp was screened, and the rejects and yield percentage were determined gravimetrically. Handsheets were then prepared and analyzed for tensile index [41].

#### 2.2.8. Statistics

Tukey’s test was applied to evaluate variability in paper properties among the produced samples. This post hoc procedure allows for multiple pairwise comparisons between group means, identifying which specific groups differ significantly once the Analysis of Variance (ANOVA) indicates an overall statistical difference among three or more groups.

## 3. Results and Discussion

### 3.1. Kraft Lignin and Synthesized Polyurethane Characterization

Understanding lignin structure is essential for its effective application in polyurethane synthesis. In this study, the industrial softwood kraft lignin employed showed an overall lignin purity of 96.51%, with acid-insoluble and acid-soluble lignin contents of 92.26% and 4.25%, respectively, and an ash content of 1.13%. These results confirm the high purity of the kraft lignin and are consistent with previous findings [46,47,48]. The remaining 2.37% was attributed to unidentified components, most likely residual carbohydrates [46]. The lignin exhibited a weight-average molecular weight (Mw) of 7956 g/mol and a number-average molecular weight (Mn) of 1073 g/mol, resulting in a polydispersity index (PDI) of 7.4, indicative of a heterogeneous molecular distribution, in agreement with the literature [46,47,49].

**Figure 1:**
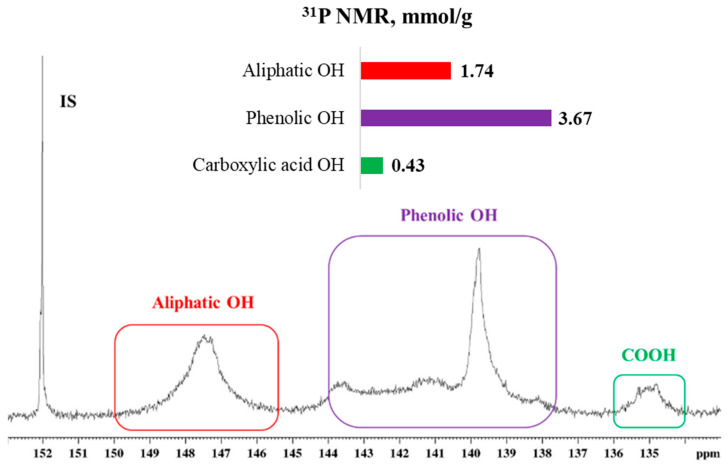
^31^P NMR spectra of softwood kraft lignin, normalized to internal standard signal at 152 ppm, with chemical shift assignments and quantification of aliphatic, phenolic, and carboxylic hydroxyl groups.

Quantitative ^31^P NMR spectroscopy enables identification and quantification of hydroxyl groups in kraft lignin, including aliphatic (145.4–150 ppm), carboxylic (133.6–136 ppm), and various phenolic moieties (137.6–144 ppm) [15,32,49]. The hydroxyl group distribution obtained in this study is consistent with values reported in the literature for softwood kraft lignin [47,49,50,51], as can be seen in Figure 1.

Characterization of the hydroxyl groups in lignin is critical for understanding and optimizing polyurethane synthesis, as these functional groups directly participate in the formation of urethane bonds. Aliphatic hydroxyl groups (1.74 mmol/g) exhibit higher nucleophilicity and reactivity with isocyanate groups, thereby enhancing reaction efficiency [52]. In contrast, phenolic hydroxyl groups (3.67 mmol/g) are less reactive toward isocyanates. To address this limitation, previous studies have proposed the use of catalysts to increase the reactivity of phenolic hydroxyls, either by accelerating reaction rates or by compensating for the limited availability of aliphatic hydroxyl groups in lignin-based formulations [53].

**Figure 2:**
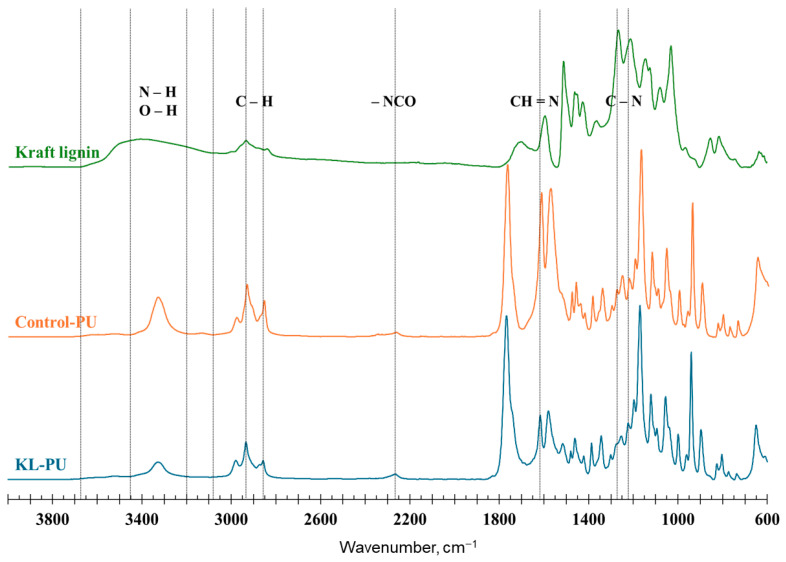
Normalized FTIR spectra of kraft lignin, polyurethane control sample (Control-PU), and lignin-based polyurethane (KL-PU). The dotted vertical lines highlight the characteristic wavenumber ranges associated with functional groups discussed in the text.

Following the hydroxyl group analysis, FTIR spectroscopy was conducted to confirm the formation of polyurethane. In the Control-PU sample (GVL-DABCO-HDI system), polyurethane formation was limited because partial opening of GVL at 105 °C [28] generated only a small number of hydroxyl groups capable of reacting with isocyanates. In contrast, the incorporation of kraft lignin significantly enhanced polyurethane formation due to its abundance of reactive hydroxyl functionalities, which promote the reaction with isocyanate groups. Figure 2 shows the normalized FTIR spectra of the kraft lignin used as polyol, the resulting lignin-based polyurethane, and the control sample synthesized without lignin.

The FTIR spectra display characteristic absorption bands associated with functional groups present in lignin and polyurethane. The broad band observed between 3700 and 3000 cm^−1^ corresponds to O–H stretching vibrations, attributable to both aliphatic and phenolic hydroxyl groups [54]. Absorption bands located at approximately 2930 cm^−1^ and 2856 cm^−1^ are assigned to asymmetric and symmetric stretching of C–H bonds in methyl and methylene groups, respectively. These bands are consistently observed in both polyurethane and lignin-based materials [55,56]. Notably, in the polyurethane spectra, the hydroxyl stretching region shifts toward lower wavenumbers (approximately 3100–3400 cm^−1^), indicating the formation of urethane N–H bonds resulting from the reaction between hydroxyl and isocyanate groups [55].

The appearance of the –NCO characteristic band near 2260 cm^−1^ suggests an excess of isocyanate groups during polymerization, indicating incomplete reaction of HDI. In some cases, a subtle residual signal in this region may reflect trace amounts of unreacted NCO or secondary products derived from excess isocyanate [55,56]. A distinct absorption band at 1767 cm^−1^ is attributed to the C=O stretching vibration of GVL, which typically exhibits a carbonyl band near 1786 cm^−1^. When interacting with hydroxyl-rich surfaces, this band tends to shift to lower wavenumbers, at approximately 1767 cm^−1^ [57]. Notably, this band partially overlaps the spectral region typically associated with urethane C=O stretching (1710–1730 cm^−1^) [25,55,56], making the urethane carbonyl not clearly distinguishable in this study.

The band at 1616 cm^−1^, characteristic of polyurethane structures, can be attributed to imine (−H=N) groups [58,59]. The 1570 cm^−1^ band is attributed to both C=C aromatic stretching and N–H in secondary amides [60]. Bands within the region of 1600 to 1510 cm^−1^ are attributed to aromatic ring vibrations, encompassing C=C stretching and C–H bending. Bands between 1265 and 1220 cm^−1^ correspond to C–N stretching in urethane linkages [61,62], while the signal near 1210 cm^−1^ is related to C–O stretching in aliphatic hydroxyl groups [54]. Further, vibrations associated with C–O–C stretching appear between 1150 and 1060 cm^−1^ [25].

**Figure 3:**
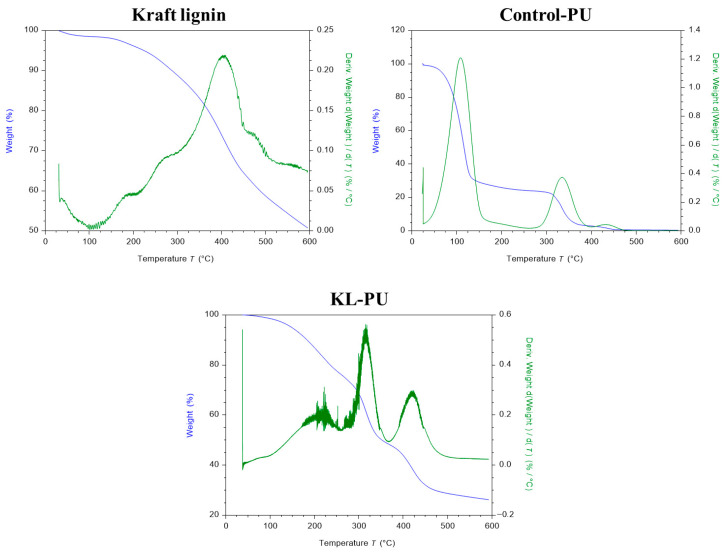
Thermogravimetric analysis (TGA) and differential thermogravimetry (DTG) profiles of kraft lignin, polyurethane control sample (Control-PU), and lignin-based polyurethane (KL-PU).

An overlap of bands in the FTIR spectra prevented unambiguous identification of some characteristic polyurethane bands. Therefore, FTIR results were interpreted as indicative of urethane bond formation rather than as standalone evidence of a fully developed polymer network. In this context, the TGA results provided more conclusive evidence, as distinct stages of thermal degradation associated with urethane bond decomposition confirmed successful polyurethane formation. Figure 3 presents the thermal performance of kraft lignin, Control-PU, and KL-PU.

The thermal degradation profile of softwood kraft lignin exhibits characteristic distinct stages, consistent with earlier work [23,48,63]. The first mass loss between 50 and 110 °C corresponds to the release of moisture and volatile components. The second major degradation event, observed between 200 and 450 °C, is associated with the cleavage of α- and β-aryl-alkyl-ether linkages, aliphatic side chains, and decarboxylation reactions [64]. At higher temperatures (525 to 600 °C), degradation is dominated by the cleavage of more stable C-C bonds. Differential thermogravimetry (DTGMax) highlights a pronounced degradation peak at approximately 400 °C, reflecting the main decomposition event of lignin [48].

The TGA of the synthesized polyurethane material reveals a distinct profile compared to kraft lignin. In the Control-PU sample, an initial and significant weight loss below 150 °C can be attributed to the evaporation of residual GVL retained in the polymer matrix, in line with FTIR evidence (Figure 2) [65]. In the KL-PU thermodegradation profile, this initial sharp weight loss can be seen from 150 °C, with maximum degradation at 221.9 °C, correlating with the loss of both GVL and HDI residues. Subsequent degradation proceeds through two main steps. The first, between 275 and 350 °C, arises from the simultaneous degradation of lignin structures and cleavage of urethane bonds, producing primary amines, olefins, carbon dioxide, and phenolic compounds [26,66]. The second, between 400 and 500 °C, corresponds mainly to the scission of ether linkages within the lignin backbone [66].

The initial degradation temperatures (T_onset_), temperatures corresponding to 50% mass loss (T_50_), the maximum degradation rate temperatures (DTG_Max_), and the residual mass at 600 °C at the end of the heating ramp are summarized in Table 2.

Comparison with the lignin-free polymer clearly demonstrates that lignin shifts both the onset temperatures and the degradation pathways, underscoring its reactive participation within the polyurethane matrix rather than acting as an inert filler. These insights highlight lignin’s active role in the polymer and establish a basis for evaluating the resulting materials as a functional barrier coating for recycled linerboard.

### 3.2. Paper Characterization

The initial evaluation of the paper samples focused on grammage and thickness (Table 3), two fundamental parameters that directly influence both barrier performance and the physicomechanical properties of the paper. Due to the limited polyurethane formation in the absence of lignin, the Control-PU formulation was not applied to paper. In contrast, the lignin-containing formulation was prioritized, as the abundance of hydroxyl groups in lignin promotes reactivity and facilitates the effective formation of polyurethane network.

**Figure 4:**
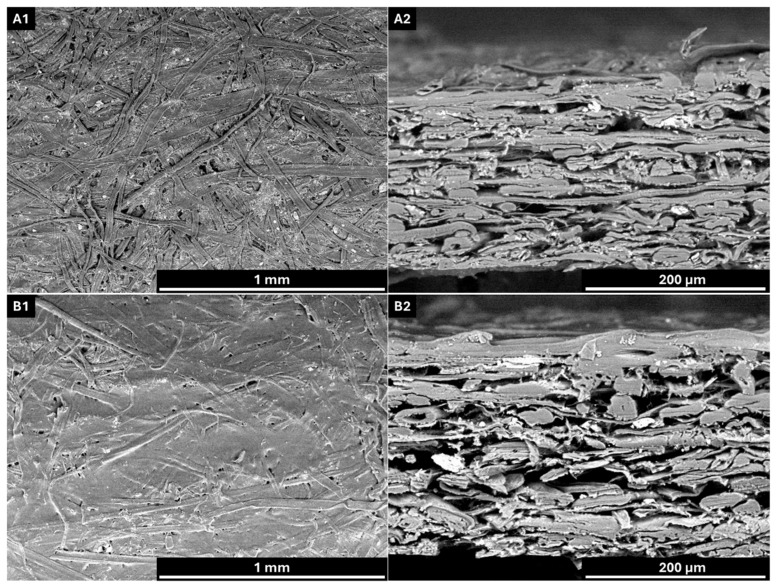
SEM images of uncoated and coated paper. (**A1**) Surface morphology of uncoated paper; (**A2**) cross-sectional view of uncoated paper; (**B1**) surface morphology of coated paper; (**B2**) cross-sectional view of coated paper. AV: 15 kV; WD: 6.5 mm. Magnification: ×100 (**A1**,**B1**) and ×400 (**A2**,**B2**).

The substrate used in this study was a recycled linerboard with a basis weight of 166.6 g/m^2^ and a thickness of 251 µm, resulting in a bulk of 1.5 cm^3^/g. These values are consistent with literature data for linerboard papers [67]. According to Tukey’s test at a 5% significance level, all grammage values were statistically different from each other. In contrast, for thickness, only the uncoated sample (linerboard) showed a significant difference compared to the coated papers, which did not differ among themselves. Although the coating increased the paper mass, it formed a relatively thin and compact layer, even across variations in coating weight. To illustrate the interaction between the coating and the paper substrate, as well as to visually confirm the formation and distribution of the coating layer, scanning electron microscopy (SEM) analyses were performed. Figure 4 presents representative images of both the paper surface and cross-section.

SEM images reveal that the coating layer is well defined upon application, forming a film over the paper surface. The surface morphology of the coated paper exhibits a more homogeneous appearance with reduced porosity compared to the uncoated sample.

#### 3.2.1. Barrier Properties

**Figure 5:**
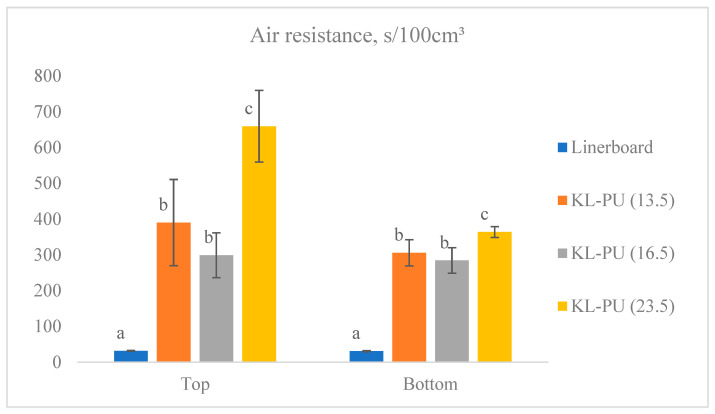
Air resistance (Gurley) of uncoated linerboard and coated samples. Different lowercase letters (a–c) indicate statistically significant differences among samples (*p* < 0.05).

Air permeability is a key parameter in evaluating the performance of paper-based packaging materials, as it directly reflects the porosity of the paper related to the fiber bonding network. High air permeability indicates a more open structure, which can compromise barrier properties and reduce protection against moisture, gases, and external contaminants. To assess the reduction in porosity, air permeability was measured using the Gurley method, a standard air resistance test, and the results are presented in Figure 5.

The uncoated linerboard exhibited an air resistance of approximately 31 s/100 cm^3^ on both sides, reflecting its inherently porous structure, and the high permeability of the paper matrix, which allows for significant airflow through the paper matrix. Such structural characteristics can promote coating penetration and affect film formation on the surface. The absorption of the coating into the paper can help fill the pores of the paper and act as an adhesive, enhancing fiber network bonding [10].

For the coated samples, the side on which testing was performed influenced the measured properties. All coated paper samples differed significantly (5% significance level in Tukey’s test) from the linerboard (uncoated), while KL-PU (13.5) and KL-PU (16.5) remained statistically similar. The gain in air resistance with the coating application ranged from 267.4 to 627.8 s/100 cm^3^, representing an improvement of approximately 8-fold compared to the uncoated paper. On the bottom side of the paper, the same statistical trend was observed, with KL-PU (13.5) and KL-PU (16.5) showing similar performance, although differences between the coated papers were less pronounced. Here, resistance gains reached a maximum of 332.9 s/100 cm^3^.

In addition to air permeability, water resistance was evaluated through water vapor transmission rate (WVTR), contact angle, and Cobb_1800_ water absorption tests. Together, these analyses provide complementary insights into vapor-phase moisture permeation, surface hydrophobicity, and the material’s barrier performance against liquid water. The WVTR and contact angle results for each sample are summarized in Table 4.

The WVTR results demonstrate a clear improvement in the barrier properties of the coated samples compared to the uncoated linerboard. The uncoated paper presented a WVTR of 28.1 ± 3.04 g/m^2^/h, consistent with values typically reported for cellulose-based materials [68]. In contrast, the coated samples exhibited a significant reduction in WVTR, with barrier improvements ranging from 24.6 to 26.8 g/m^2^/h. Notably, the KL-PU (23.5) sample, which had the highest coating grammage, achieved the lowest WVTR (1.3 g/m^2^/h), demonstrating that increasing coating weight minimizes structural features, such as micropores.

Regarding the contact angle, the uncoated linerboard showed the highest value (103.6 ± 3.4°). After coating, a decrease in the contact angle was observed, with values between 83.4° and 85.8°, suggesting a more hydrophilic surface. The contact angle reflects the initial wetting behavior at the surface and is primarily governed by surface chemistry. In contrast, WVTR is controlled by the bulk structure of the coating and its ability to hinder vapor diffusion through the paper. This behavior is likely related to the chemical nature of the polyurethane matrix [69], which, despite providing excellent barrier performance against water in both liquid and vapor form, contains polar moieties that may reduce surface hydrophobicity. Lignin further contributes to this effect through its abundant polar functional groups, such as hydroxyl and carboxyl groups, imparting additional hydrophilicity to the polymer network [70].

**Figure 6:**
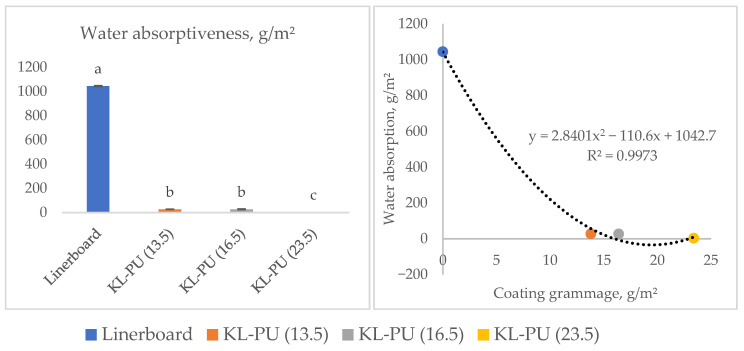
Water absorptiveness (Cobb_1800_) of uncoated and coated linerboard samples. Different lowercase letters (a–c) indicate statistically significant differences among samples (*p* < 0.05).

While the contact angle provides valuable insight into the surface hydrophobicity of the coated papers, it only reflects the initial resistance to water penetration at the interface. To complement the water resistance tests, the Cobb test was performed to quantify the total water absorption capacity over a defined period, providing a direct measure of the coating’s effectiveness in reducing liquid water uptake through the bulk structure of the paper. The test was carried out over 1800 s, and the results are shown in Figure 6.

The Cobb_1800_ results demonstrated a substantial decrease in water absorption for all coated samples, with reductions ranging from 1017 to 1041 g/m^2^ compared to the uncoated linerboard. According to the Tukey test at a 5% significance level of coated samples, KL-PU (23.5) was statistically different. Nonetheless, the KL-PU (23.5) sample, which had the highest coating weight, exhibited the lowest Cobb value (3.4 g/m^2^), while the other coated samples presented values around 27.4 g/m^2^. The application of the coating reduced the Cobb value 37- to 307-fold compared to the uncoated linerboard.

These significant reductions in water absorption observed through the Cobb test demonstrate the coating’s effectiveness in limiting liquid water uptake, which is critical for improving both the dimensional stability and mechanical integrity of paper when exposed to moisture. Water molecules penetrating the paper matrix interact with hydroxyl groups in cellulose, leading to fiber swelling and a consequent reduction in the internal strength of paper [71].

Despite improvements in water absorption and vapor permeation resistance, the surface did not reach a highly hydrophobic state, as evidenced by the moderate contact angle values of the coated linerboards. In paper-based applications, water absorption is generally considered a more reliable indicator of water resistance than contact angle measurements. Although the contact angle reflects surface wettability, it does not fully represent how water penetrates the material. In cellulose pulps, absorption is primarily governed by the internal structure and chemistry rather than surface hydrophilicity [72]. Similarly, in polyurethanes, bulk water uptake is influenced by the morphology of the polymer network and the distribution of polar groups, not solely by surface wettability [73].

**Figure 7:**
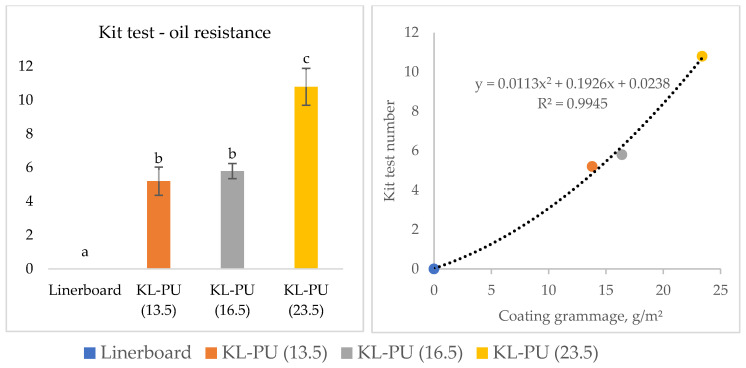
Grease and oil resistance test of uncoated and coated linerboard samples. Different lowercase letters (a–c) indicate statistically significant differences among samples (*p* < 0.05).

To complete the barrier characterization, oil and grease resistance tests were conducted to assess the coating’s ability to block non-aqueous substances. These substances can exhibit varying penetration behaviors due to their distinct chemical and physical properties, depending on papers characteristics [74]. The Kit test results are presented in Figure 7.

The uncoated linerboard exhibited a Kit rating of 0, indicating complete absorption of the most viscous oil used in the test and, consequently, no barrier against grease and oil. In contrast, the application of KL-PU coatings markedly improved oil resistance, with the samples achieving Kit ratings of 5, 6, and 11 for KL-PU (13.5), KL-PU (16.5), and KL-PU (23.5), respectively. According to food packaging industry requirements, an oil resistance level between 6 and 9 is generally sufficient to meet the demands of most applications involving oily or fatty foods [75]. Higher Kit ratings reflect the formation of a more continuous and less porous coating layer, effectively preventing the diffusion of non-polar substances through the paper matrix.

#### 3.2.2. Mechanical Properties

**Figure 8:**
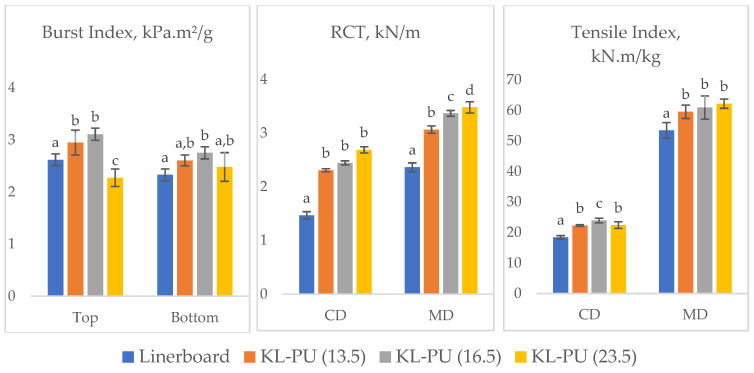
Mechanical performance of uncoated and coated linerboard samples: Burst Index (top and bottom sides), RCT and Tensile Index, in cross direction (CD) and machine direction (MD). Different lowercase letters (a–d) indicate statistically significant differences among samples (*p* < 0.05).

In addition to barrier performance, the evaluation of mechanical performance is essential to ensure that coated linerboards maintain adequate strength for handling, processing, and end-use applications. To this end, the burst index, ring crush test (RCT), tensile index, elongation, and elasticity modulus were measured to assess the impact of the coating on the physical integrity of the paper. Figure 8 presents the results of these tests.

In general, the application of lignin-based polyurethane coatings influenced the mechanical behavior of the linerboard in distinct ways, depending on the type of mechanical stress and fiber direction. Regarding the burst index, a property closely related to the integrity of the fiber network and inter-fiber bonding, a reduction was observed at the highest coating weight. This decline may be attributed to increased absorption of the polymer into the paper structure, as suggested by the thickness measurements, which may have disrupted fiber-to-fiber contact and altered stress distribution within the coated paper. In addition, the formation of a stiffer polymer phase, formed by the lignin addition in the polyurethane matrix [76,77], can reduce the ability of the fiber network to undergo out-of-plane deformation, which is critical for resisting multiaxial stresses during the burst test.

In contrast, RCT, which measures compressive strength, showed a clear improvement with increasing coating grammage. This behavior can be explained by the fact that RCT depends not only on fiber bonding but also on stiffness and resistance to buckling, both of which are reinforced by the polymer layer, acting as a protective shell around the paper matrix.

For the tensile index, the application of the coating improved performance compared to the uncoated sample. Nevertheless, no significant differences were observed among the different coating weights, suggesting that a threshold was reached beyond which additional coating does not contribute further to tensile strength. This likely reflects the balance between gains in stiffness and losses in inter-fiber bonding.

Although both burst index and tensile strength are related to fiber–fiber bonding, they are governed by different failure mechanisms. Burst strength is more sensitive to network flexibility and out-of-plane deformation, whereas tensile strength is mainly controlled by in-plane load transfer and stress distribution along the sheet. Thus, the formation of a continuous and relatively rigid lignin-based polyurethane coating can enhance tensile strength under uniaxial loading, even if partial polymer penetration locally disrupts fiber–fiber contacts and reduces burst resistance.

Comparable trends have been reported by Wu et al. (2022) [25], who demonstrated that incorporating lignin nanoparticles (LNPs) into polyurethane matrices enhanced tensile strength and elongation through hydrogen bonding and void-filling, enabling better dissipation of mechanical energy. However, at higher loadings, the aggregation of LNsP led to structural defects, negatively impacting mechanical performance [25]. A similar mechanism may explain the mechanical behavior observed in this study.

### 3.3. Biodegradation of Coated and Uncoated Linerboard

**Figure 9:**
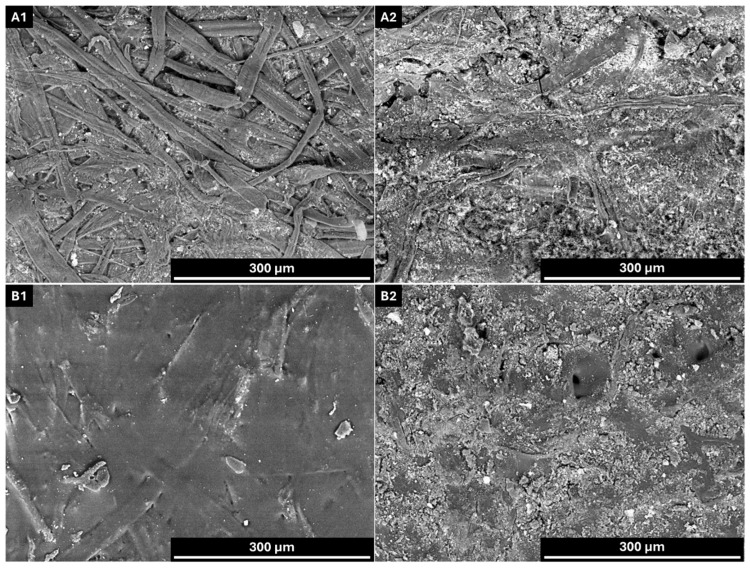
Scanning electron microscopy (SEM) images of uncoated and coated linerboard samples before (**A1**,**B1**) and after (**A2**,**B2**) 42 days of soil burial. (**A1**,**A2**) Uncoated; (**B1**,**B2**) KL-PU (23.5). AV: 15 kV; WD: 6.5 mm; Magnification: ×300.

Biodegradation was assessed through a soil burial test conducted at 58 °C and 52% relative humidity over 42 days. The biodegradation rate was determined based on the mass loss of each material over time. Since the same coating formulation was applied across all coated samples, the one with the highest coating load, KL-PU (23.5), was selected as a representative for this analysis. At the end of the testing period, the uncoated linerboard exhibited a mass loss of 52.48%, whereas the coated sample, KL-PU (23.5), showed a mass loss of 37.47%. These results indicate that the coating conferred a certain resistance to biodegradation, likely due to reduced water permeability and slower microbial attack on the coated surface. Although the overall degradation of the coated paper was limited, evidence of polyurethane degradation was observed, consistent with the behavior reported by Feng et al. (2019) for vegetable oil-based PUs [78]. Before and after the biodegradation test, the papers were analyzed by SEM to illustrate surface degradation during the test, as shown in Figure 9.

At the end of the testing period, the paper samples exhibited pronounced morphological alterations, characterized by the collapse of their original structure and a noticeable increase in brittleness.

### 3.4. Repulpability of Coated and Uncoated Linerboard

The evaluation of repulpability and recyclability is essential when coatings are applied to paper, as it assesses both fiber recovery efficiency and the mechanical integrity of the recycled material. As with the biodegradation test, the sample with the highest coating weight, KL-PU (23.5), was selected for this analysis.

**Figure 10:**
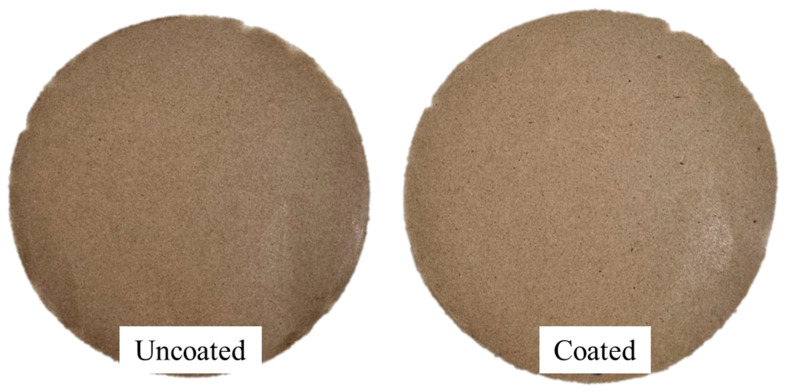
Handsheets produced after repulpability test.

The repulpability results demonstrated a significant reduction in fiber recovery yield for KL-PU (23.5), achieving only 72.1%, compared to 99.7% for the uncoated linerboard. During disintegration, some fibers remained bonded to the coating matrix, hindering their separation. Once applied, polymer coatings adhere strongly to paper surfaces, limiting recycling efficiency and natural degradation [79]. Additionally, a decrease in the tensile index of the repulped handsheets was observed. The handsheets from the uncoated sample exhibited a tensile index of 18.97 kN/kg, whereas those from the coated sample showed 15.14 kN/kg, corresponding to an approximate 20% reduction. Despite these differences, no significant visual alterations were detected in the handsheets, as illustrated in Figure 10.

These results highlight a clear trade-off between improved barrier properties and end-of-life recyclability. While the KL-PU (23.5) coating effectively enhanced the functional performance of the linerboard, its reduced repulpability indicates a potential limitation for conventional recycling processes. The strong adhesion between the coating and the fiber network prevents efficient fiber liberation during disintegration, thereby compromising fiber recovery yields and the mechanical strength of recycled products. As an alternative recycling strategy, Wang et al. (2025) [79] proposed the use of organic solvents to dissolve coatings, achieving complete removal of polymer layers from kraft paper surfaces. If optimized, this approach could be adapted for the lignin-based polyurethane developed in this study, allowing for the recovery of the coating and its possible reuse [79].

## 4. Conclusions

In this study, industrial softwood kraft lignin solubilized in γ-valerolactone (GVL) was successfully employed to synthesize polyurethane for paper coating applications. The formation of a well-structured polymeric network was confirmed by FTIR and TGA analyses, consistently demonstrating the establishment of characteristic chemical bonds. A key innovation of this work lies in the combined use of industrial lignin and GVL to produce a lignin-based polyurethane capable of forming a continuous coating layer with effective barrier performance, demonstrating the technical feasibility of this system for packaging paper applications.

Coating weight had a significant impact on the functional performance of the linerboard. As coating grammage increased, substantial improvements were observed in water barrier properties, particularly in water vapor transmission rate (WVTR) and Cobb_1800_ values. Regarding oil resistance, the coating demonstrated excellent performance, achieving a Kit rating of 11 at the highest grammage. Enhancements were also verified in mechanical performance, notably in ring crush (RCT) and tensile strength.

Biodegradation assessments revealed that, despite the presence of the coating, the material maintained gradual degradability under soil burial conditions. However, repulpability tests indicated a reduction in fiber recovery efficiency, suggesting that while the coating improved barrier and mechanical properties, it imposed some constraints on recyclability. Overall, the lignin-based polyurethane coating represents a promising and more sustainable alternative for improving the functional properties of recycled linerboard, effectively balancing enhanced material performance with environmental considerations.

## Figures and Tables

**Table 1:** Procedure Standards analyzed.

Procedure	Standard
Grammage of Paper and Paperboard	TAPPI/ANSI T 410 om-23 [33]
Thickness (caliper) of paper, paperboard, and combined board	TAPPI/ANSI T 411 om-21 [34]
Air resistance of paper (Gurley method)	TAPPI/ANSI T 460 om-21 [35]
Water absorptiveness of sized (non-bibulous) paper, paperboard, and corrugated fiberboard (Cobb test)	TAPPI/ANSI T 441 om-20 [36]
Grease resistance test for paper and paperboard	TAPPI T 559 cm-22 [37]
Water vapor transmission rate	ASTM E96/E96M-21 [38]
Contact angle	TAPPI T 458 cm-14 [39]
Ring crush strength was tested using the rigid support method	ANSI/TAPPI T 822 om-22 [40]
Tensile properties	TAPPI/ANSI T 494 om-22 [41]
Burst strength of linerboard	TAPPI T 807 om-16 [42]

**Table 2:** Thermal properties determined by TGA.

Sample	T_onset_ (°C)	T_50_ (°C)	Residues_600°C_ (%)	DTG_Max_ (°C)
KL	224.1	595.6	50.8	399.4
Control-PU	66.7	116.2	0.4	115.4
KL-PU	245.8	352.3	26.2	315.3

**Table 3:** Mean values and standard deviations of grammage and thickness for uncoated and coated papers. Different lowercase letters (a–d) indicate statistically significant differences among samples (*p* < 0.05).

Sample	Grammage, g/m^2^	Thickness, µm
Linerboard	166.6 ± 0.8 a	251 ± 3 a
KL-PU (13.5)	180.6 ± 1.2 b	263 ± 5 b
KL-PU (16.5)	183.5 ± 1.1 c	263 ± 3 b
KL-PU (23.5)	191.1 ± 1.4 d	267 ± 4 b

**Table 4:** Water Vapor Transmission Rate (WVTR) and Contact Angle of uncoated and coated linerboard samples. Different lowercase letters (a–c) indicate statistically significant differences among samples (*p* < 0.05).

Sample	WVTR, g/m^2^/h	Contact Angle, °
Linerboard	28.1 ± 3.04 a	103.6 ± 3.4 a
KL-PU (13.5)	3.1 ± 0.25 b	85.8 ± 1.3 b
KL-PU (16.5)	3.5 ± 0.47 b	84.6 ± 4.6 b
KL-PU (23.5)	1.3 ± 0.02 c	83.4 ± 3.7 b

## Data Availability

The raw data supporting the conclusions of this article will be made available by the authors on request.

## Abbreviations

The following abbreviations are used in this manuscript:

KL	Kraft Lignin
RED	Relative Energy Difference
LNPs	Lignin nanoparticles
PU	Polyurethane
GVL	Gamma-valerolactone
FTIR	Fourier Transform Infrared Spectroscopy
TGA	Thermogravimetric Analysis
GPC	Gel Permeation Chromatography
^31^P NMR	Phosphorus Nuclear Magnetic Resonance
SEM	Scanning Electron Microscopy
DABCO	1,4-diazabicyclo[2.2.2]octane
HDI	Hexamethylene diisocyanate
KL-PU	Kraft lignin-based polyurethane
Control-PU	Gamma-valerolactone -based polyurethane
